# Lupus thrombocytopenia: pathogenesis and therapeutic implications

**DOI:** 10.31138/mjr.28.1.20

**Published:** 2017-03-28

**Authors:** Nikolaos Galanopoulos, Anna Christoforidou, Zoe Bezirgiannidou

**Affiliations:** 1 Outpatient Department of Rheumatology, University General Hospital of Evros (Alexandroupolis), Thrace, Greece; 2 Department of Haematology, Democritus University of Thrace, Alexandroupolis, Greece

**Keywords:** systemic lupus erythematosus, thrombocytopenia, mycophenolate mofetil, rituximab, thrombopoietin receptor agonists

## Abstract

Systemic Lupus Erythematosus (SLE) is frequently complicated by cytopenias. Thrombocytopenia is usually non severe and its frequency ranges from 20% to 40%. It is mostly an autoimmune process caused by autoantibodies against platelet surface glycoproteins and it is associated with worse prognosis in SLE. It can also be a result of SLE treatment with azathioprine, methotrexate and rarely hydroxychloroquine or thrombotic microangiopathy or macrophage activation syndrome. If thrombocytopenia is mild (>50×10^9^/L) and there is no other evidence of disease there is no need of therapy. Severe thrombocytopenia is less frequent and needs therapeutic management. Corticosteroids are the cornerstone of therapy. Continuous high dose oral prednisolone or pulse high dose methylprednisolone (MP) with or without intravenous immune globulin are used in the acute phase. Second line agents (hydroxychloroquine, danazol, azathioprine, cyclosporine, mycophenolate mofetil, cyclophosphamide, rituximab) are usually needed. Splenectomy is indicated for recurrent or resistant cases. There are no evidence-based guidelines to facilitate selection of one drug over another but certainly the co-existence of other systemic SLE manifestations must be taken into account. Newer therapies are emerging although there is no consensus on the treatment of refractory lupus thrombocytopenia due to the absence of controlled randomized trials.

Systemic Lupus Erythematosus (SLE) is a systemic autoimmune disorder that is frequently complicated by hematological manifestations such as hemolytic anemia, leukopenia and thrombocytopenia.^1,2^ These disorders are included in the diagnostic criteria of SLE both in the earlier (1982), as well as in the revised criteria of the American College of Rheumatology (2003), and the 2012 Systemic Lupus International Collaborating Clinics (SLCC) criteria.^3–5^

Thrombocytopenia (<100×10^9^/L) has been reported in 20% to 40% of patients with SLE^2,6–8^ and is usually attributed to an autoimmune mechanism similar to that of idiopathic immune thrombocytopenia (ITP). It may be the first manifestation of lupus in up to 16% of patients, presenting months or as early as 10 years before diagnosis.^9,10^ It can also be a complication of therapeutic agents such as azathioprine, methotrexate and rarely hydroxychloroquine. Severe thrombocytopenia (<20 to 50×10^9^/L according to study definition) is relatively rare, occurring in about 3–10% of patients.^11,12^ Occasionally, thrombocytopenia may be due to the development of thrombotic microangiopathy,^13,14^ antiphospholipid syndrome or splenomegaly with hypersplenism. Macrophage activation syndrome should be suspected in patients with multiple cytopenias and fever with a rapid onset especially those with juvenile SLE.^15^

Thrombocytopenia in SLE is associated with a worse prognosis and higher mortality from the disease.^12,16,17^ It has been linked with a severe disease course including neuropsychiatric disorders, renal involvement, hemolytic anemia and antiphospholipid syndrome.^7,18^ Most studies have shown an association between thrombocytopenia and mortality, however a few did not found such a relationship.^1,19^ In two large studies thrombocytopenia was the only independent predictor for mortality in SLE.^16,20^

The leading cause of death in the Reveille et al.^20^ study was infection. It has been shown that it is not thrombocytopenia itself that influences survival, but its coexistence with multiple organ damage and treatment related complications.^12^ A different presentation with a less severe, chronic form of thrombocytopenia, not related to disease activity is also common, and appears to be less responsive to corticosteroid therapy.^1^

SLE has a significant genetic predisposition.^21^ In a prominent study by Scofield RH et al., families with even one member with thrombocytopenic SLE seemed to be repeatedly affected by a serious clinical phenotype that was assigned to a familial form of the disease, associated with genetic linkages at 1q22–23 and 11p13.^22^ In these family pedigrees, even the non-thrombocytopenic SLE patients presented with multiple organ damage such as serositis, nephritis, neuropsychiatric disease, and hemolytic anemia.

## PATHOGENESIS

Autoantibodies targeting antigenic glycoproteins on the platelet membrane are central to the destruction of platelets in SLE.^23–25^ In some cases other autoantibodies such as antiphospholipid antibodies^26,27^ and autoantibodies against thrombopoietin (TPO) or TPO receptor (c-mpl) are identified.^28–30^ Antibody-coated platelets are subsequently removed by splenic and other reticular macrophages, through binding on their surface Fc gamma receptor.

Anti-IIb/IIIa antibodies, either circulating or platelet-bound (MAIPA method), are the most frequent finding, like in ITP, but similar to ITP they are not specific for thrombocytopenia, as their detection ranges from 30–70% in thrombocytopenic patients and in contrast many patients positive for these antibodies do not ever develop thrombocytopenia.^31,32^ However, in previously antibody-positive patients recovering from thrombocytopenia after immunosuppressive therapy, these antibodies disappear and reappear in relapses, thus indicating their pathogenetic role.^23, 25^ Antibodies against Gp Ia/IIa, HLA I and Gp Ib/Ix complex are less frequently detected.

Serum levels of TPO are higher in thrombocytopenic SLE patients compared to normal controls, and megakaryocytes are increased in their bone marrow. Nevertheless, antibodies against TPO and c-mpl have been identified in 23%–39% of patients.^25,28^ These patients often have lower platelet counts, although their exact role in the pathogenesis of thrombocytopenia remains obscure. In some cases the development of anti-TPO antibodies is associated with poor response to administration of corticosteroids (CS).^30^

Antiphospholipid antibodies against cardiolipin, phosphatidylinositol, prothrombin as well as lupus anticoagulant are detected in a substantial proportion of patients with SLE and thrombocytopenia^31–33^ and they are significantly associated with thrombocytopenia in many studies.^34,35^ Membrane phospholipids that cross-react antigenically with cardiolipin are exposed after cell damage and comprise the trigger for the development of anti-cardiolipin antibodies.^36^

Finally, autoantibodies against the CD40-ligand molecule on the surface of T lymphocytes appear to have a role in the development of thrombocytopenia in SLE. CD40-ligant is linked to the CD40 antigen on the B lymphocytes surface leading to their activation and autoantibody production. In a study by Nakamura et al.^37^ such autoanti-bodies were detected in seven (6%) of 125 patients with SLE. The incidence of thrombocytopenia was higher in positive patients in comparison to the negative ones (100% vs 14%).

## LABORATORY FINDINGS

Laboratory investigation of patients with SLE and thrombocytopenia begins with microscopic examination of peripheral blood smears for the estimation of platelet count and size and the presence of schistocytes (fragmented red blood cells). In case of coexistence of thrombocytopenia and anemia the assessment of reticulocyte count and direct Coombs test is warranted. Abnormal lymphocyte morphology as well as lymphadenopathy should raise suspicion of a lymphoproliferative disease and prompt investigation with imaging studies, immunoglobulin measurement, bone marrow and lymph node biopsy. Coagulation studies, LDH, bilirubin levels and antiphospholipid antibodies are also important to exclude TTP, disseminated intravascular coagulation and antiphospholipid syndrome.

The measurement of antiplatelet antibodies may be required in patients with severe thrombocytopenia, especially in prednisone refractory ones. It should however be noted that failure to detect them does not preclude autoimmune thrombocytopenia in patients with SLE. This fact along with the limited availability of these tests has made their usefulness controversial.

High antibody titers against double-stranded DNA (anti-dsDNA) coupled with low levels of C3 and C4 advocate activity of SLE, although thrombocytopenia itself has not been found to be associated with anti-dsDNA.^11^ On the other hand low levels of C3 or CH50 has been associated with thrombocytopenia.^12^

Bone marrow aspiration may be needed in severe or persistent thrombocytopenia, particularly when there is suspicion of drug toxicity or hemophagocytosis or when it is accompanied by other cytopenias. Bone marrow findings are heterogeneous between studies, ranging from hypo- to hypercellular marrow and low, normal or high megakaryocyte number. Increased reticulin proliferation, lymphocytosis, plasmacytosis and morphological abnormalities of megakaryocytes notably aggregation, reduced lobulation and pycnotic appearance, as well as necrotic lesions in the bone marrow stroma have been reported.^25, 38^

## THERAPEUTIC MANAGEMENT

Thrombocytopenia in SLE may be acute in onset and extremely severe. Severe thrombocytopenia requires emergency therapy in order to eliminate hemorrhagic complications and achieve a complete or partial platelet response. Maintenance treatment is usually needed to prevent relapse. Initial treatment does not differ from ITP. The decision to treat when thrombocytopenia is the sole disorder in SLE depends on the hemorrhagic manifestations and platelet count. Generally, patients with a platelet count > 50×10^9^/l without bleeding manifestations do not require treatment in the absence of coexistent hemostatic disorders, anticoagulation treatment, trauma or major surgery.

Corticosteroids are the cornerstone of initial treatment. High dose oral prednisolone or pulse high dose methylprednisolone (MP) with or without intravenous immune globulin (IVIG) are used in the acute phase. Second line agents include hydroxychloroquine (HCQ), danazol, immunosuppressive drugs like azathioprine (AZA), cyclosporine (CSA), mycophenolate mofetil (MMF), cyclophosphamide (CYC), and biological therapies such as rituximab. The thrombopoietin receptor agonists romiplostim and eltrombopag have been widely used for the treatment of ITP and they also seem to have a role in SLE thrombocytopenia. Splenectomy is indicated for recurrent or resistant cases. It should be noted that none of the above agents have been tested in a randomized fashion in the context of SLE thrombocytopenia.

In a recent study, Jin-Hee Jung et al.^39^ retrospectively examined the clinical characteristics and prognosis of 230 patients with SLE in regard to degree of thrombocytopenia and response to treatment. They found higher mortality (15% vs 9%) among patients with severe thrombocytopenia (<20×10^9^/l) and lower mortality in patients with complete remission of thrombocytopenia after treatment. No difference between therapeutic modalities was found, regarding complete remission rate, except a lower incidence of complete remission in the danazol group. Similarly, Ziakas et al. performed a case-control study in 632 patients and did not find a different relapse free interval between patients receiving low or high intensity regimens (CS plus AZA vs CS plus CYC).^12^

Other causes of thrombocytopenia beyond auto-immune will not be further discussed in the below section.

## FIRST LINE THERAPY

### Corticosteroids

Corticosteroids (CS) are administered at the conventional dose of 1–1.5 mg/kg of prednisone qd with subsequent slow tapering after achieving a stable platelet response or as pulses of methylprednisolone (MP) iv (500–1000 mg/day for 3 days). There is no significant advantage of the higher dose MP scheme (3–5g), which may be associated with more complications like infections.^40^ Anti-TPO receptor antibodies may be associated with a suboptimal response to CS.^30^ CS appear to produce a satisfactory response in the majority of patients, but eventually most patients relapse.^2,41^ If there is no response after four weeks of CS therapy the patient is considered resistant and a second line treatment is sought.

### Intravenous Immune Globulin (IVIG)

IVIG is used in the acute management of the bleeding patient with severe SLE thrombocytopenia at a dose of 1g/kg for 1 to 2 days, as recommended in the recent 2011 American Society of Hematology guidelines for ITP^42^ or at 400mg/kg for five consecutive days.^43^ It exerts its action by downregulating autoantibody production, neutralization of pathogenic autoantibodies by anti-idiotypic antibodies, inhibition of complement-mediated damage, modulation of cytokine production, induction of apoptosis in lymphocytes, and modulation of both B- and T-lymphocyte function. Maintenance of remission after repeated lower monthly doses has also been reported.^44^ It is safely used in pregnancy.

## SECOND LINE THERAPY OR STEROID-SPARING AGENTS

Although most patients initially respond to CS, responses are not sustained and eventually management calls for second line agents. Alternative treatment is also warranted in responding patients who experience debilitating side effects from prolonged CS use. These drugs are used alone or in combination with CS as steroid-sparing agents. There are no evidence-based guidelines to facilitate selection of one drug over another but certainly the co-existence of other systemic SLE manifestations must be taken into account in order to select the appropriate therapy. If thrombocytopenia is mild (>50×10^9^/L) and there is no other evidence of disease there is no need of therapy.

### Hydroxychloroquine (HCQ)

A combination of HCQ and prednisone is effective in many patients.^41^ Khellaf et al.^45^ studied the effect of HCQ in either SLE or only ANA positive patients with thrombocytopenia and insufficient response to CS alone. The coadministration of CS with HCQ resulted in an overall response rate of 60% with a higher rate noted in patients with SLE compared to those with ANA only (83% vs 50%). Arnal et al. reported on the long-term results of HCQ plus CS in 11 patients failing CS alone, in 64% of whom a lasting response was obtained.^41^

### Danazol

Danazol is a synthetic androgen with a proven activity in many types of thrombocytopenia including that of SLE.^46,47^ Its mechanism of action is unclear but involves impairment of macrophage-mediated clearance of antibody-coated platelets via decreased Fc receptor expression. It is a well tolerated drug, with the main side effects being hirsutism and an increased risk of thromboembolic episodes. The best responses have been observed after prolonged administration at a daily dose of 600–800mg, alone or in combination with AZA or CS. Following a stable response for at least 12 months the dose can be lowered or interrupted as it has been associated with lasting responses even after discontinuation.^48^

### Azathioprine

AZA is a steroid-sparing agent with sparse reports on lupus thrombocytopenia, used alone or in combination with CS.^49,50^ It is administered at a dose of up to 2mg/kg/day. If a response occurs, therapy should be continued at full doses for at least 12 months and then tapered gradually.

### Cyclosporine

In a small number of patients with SLE and thrombocytopenia CSA appeared to be beneficial as a second line or steroid-sparing agent.^51,52^ The optimal dose has not been defined and patients may respond in doses much lower than those used in transplantation (<3–5mg/kg/day). However, caution should be used because of its renal toxicity.

### Cyclophosphamide

Cyclophosphamide is of particular importance in the treatment of severe refractory thrombocytopenia of SLE.^52^ It is administered in IV pulses (0,75–1 g/m^2^ or 10–15 mg/kg every 4 weeks for four to six months). In the retrospective study of Boumpas et al.^53^ in 7 patients with SLE and thrombocytopenia refractory to CS, CYC at a dose of 0,75–1 g/m^2^ every 4 weeks resulted in all patients achieving CR within 2–18 weeks. In four patients the subsequent administration of low dose prednisolone was sufficient to maintain remission of thrombocytopenia throughout a long follow up period (mean 5.6 years). Alternatively, Park et al.^54^ applied the low dose CYC protocol (500 mg of CYC every two weeks for three months), as used in the Euro-Lupus Nephritis Trial, in 2 refractory patients achieving a good response which was maintained thereafter by a combination of low dose prednisolone with AZA or MMF. This lower dose can improve tolerability of CYC by reducing the risk of neutropenia.

### Mycophenolate mofetil

MMF, a prodrug of mycophenolic acid, which inhibits inosine-5’monophosphate dehydrogenase, is an immunosuppressive drug successfully administrated both as treatment of severe resistant thrombocytopenia and as maintenance.^55,56^ Its main use has been lupus nephritis and it is less toxic than CYC.

### Splenectomy

Splenectomy has been successfully performed is SLE thrombocytopenia.^57^ This procedure has been widely used, with excellent long-term results in recurrent ITP.^58^ However, in SLE there is a risk of a flare of the disease because the spleen is a prominent site of immune complexes’ clearance, and additionally a predisposition to infections due to the often prolonged use of immunosuppressants in these patients.^1^ Prophylactic vaccinations against pneumonococcus sp, haemophilus influenza type B and meningitis are mandatory before the procedure and antibiotic prophylaxis for at least two years postsplenectomy are recommended, particularly in patients with additional chronic hypocomplementemia. Efficacy of splenectomy is controversial in SLE^59^ with one study reporting only 2 out of fourteen patients maintaining response without the need of CS or other drugs.^60^ Thus, it is reserved for the most resistant cases. More studies are needed to define the safety and efficacy of the procedure.

## NOVEL THERAPIES

### Rituximab

SLE is known to be associated with polyclonal B-cell hyper reactivity. Rituximab is a chimeric monoclonal antibody that binds to the CD20 antigen, found on the surface of B lymphocytes, inducing depletion of B cells. In SLE, it has been used at high doses of 1000 mg for one or multiple doses to as low as 100 to 200mg per week for 1–4 doses,^61–63^ the lower doses reserved for patients with cytopenias only.^64^ Although retrospective or open label studies and case reports have shown efficacy over 60% in lupus thrombocytopenia, optimal dose has not been defined and duration of response is somehow short lived.^61,65^ Newer, type II, anti-CD20 mAbs like obinotuzumab may be more effective in this context due to more potent B cell depletion.^66^ On the other hand, results from two randomized controlled studies (EXPLORER^67^ and LUNAR^68^ trials) using rituximab in an intention-to-treat SLE have been disappointing^69^ showing no significant benefit over placebo showing no significant benefit over placebo in BILAG scores and renal response respectively. This unexpected outcome has been partially attributed to suboptimal trial design and concurrent therapies, but it has also been speculated that a feedback effect, characterized by rising BAFF levels, may play a role to postrituximab SLE flares.^69^ The exact place of rituximab, regarding its use early or late in the course of thrombocytopenia remains to be defined by further studies.

### Belimumab

Belimumab is a human IgG1λ monoclonal antibody that binds to and inhibits B-cell activating factor (BAFF), thus inhibiting the biological activity of B lymphocytes and inducing apoptosis of autoreactive B lymphocytes.^70^ In SLE BAFF is overexpressed, enhancing the survival of B lymphocytes, including autoreactive B cell populations.^71^ In the post hoc analysis of two Lupus phase III randomized trials, belimumab was significantly associated with less worsening in the haematological domain, but there are currently no trials of belimumab for immune thrombocytopenia or hematological disorders in general.^70^

## THROMBOPOIETIN RECEPTOR AGONISTS

Romiplostim and eltrombopag are thrombopoietin receptor agonists which exhibit their action by inducing proliferation and differentiation of megakaryocyte progenitors, maturation of megakaryocytes and increased platelet production.^72^ They have displayed excellent results in phase III randomized trials^73,74^ and have been approved since 2008 as second line agents for ITP refractory to other treatments. So far, the efficacy of these agents in lupus thrombocytopenia has only been assessed in sparse case reports. In one report a patient with Evans syndrome refractory to rituximab was successfully managed with romiplostim,^75^ whereas in another case romiplostim facilitated response in a pregnant thrombocytopenic patient with SLE.^76^ Contrasting to these results a young SLE patient developed thrombotic microangiopathy with renal failure after romiplostim therapy.^77^ Maroun et al.^78^ reported on three patients with SLE thrombocytopenia in which administration of eltrombopag was successful as steroid-sparing treatment. Another patient with refractory thrombocytopenia achieved a complete response after eltrombopag treatment.^79^ Eltrombopag was also efficient in a patient with lupus-associated amegakaryocytic thrombocytopenia.^80^ The thrombotic risk must be taken into account though. In a recent report, a patient with antiphospholipid syndrome presented with fatal thrombotic complications one month after starting eltrombopag for severe thrombocytopenia and while platelets had normalized.^81^

## CONCLUSION

Thrombocytopenia (<100×10^9^/L) has been reported in 20% to 40% of patients with SLE and is usually attributed to an autoimmune mechanism similar to that of idiopathic immune thrombocytopenia. It may be the first manifestation of lupus in up to 16% of patients, presenting months or as early as 10 years before diagnosis. Many studies have found an association between thrombocytopenia and a higher mortality from SLE, although the pattern of thrombocytopenia (severity, onset) is not clearly defined. There are no evidence-based recommendations for the treatment of SLE thrombocytopenia due to the absence of randomized controlled trials. Acute therapy of severe thrombocytopenia or for the bleeding patient is the same as in ITP, high-dose CS and IVIG-based, but maintenance usually calls for the addition of other immunosuppressive drugs (hydroxychloroquine, danazol, azathioprine, cyclosporine, mycophenolate mofetil, cyclophosphamide), because response is often short-lived and patients have other organ involvement too. New drugs such as monoclonal antibodies and thrombopoietin receptor agonists are emerging as steroid-sparing modalities but safety and efficacy have yet to be established. Randomized controlled trials are needed to define the superiority of any second-line agent over the others.
